# Editorial

**DOI:** 10.1155/S1463924683000425

**Published:** 1983


Journal of Automatic Chemistry, Volume 5, Number 4 (October–December 1983), page 169

### Editorial

### Robots replacing micros as the latest buzz-word in automation?

Analytical chemistry like most subjects in modern life has a tendency to follow fashions. Gas chromatography, in vogue in the 1960s, was replaced by HPLC as the fashionable technique in the early 70s. Microcomputer applications predominated from the middle to late 70s and now the 80s threatens to be the province of the laboratory robot. However, despite the obvious lessons available to us from the previous introductions of new ideas, it would appear that many groups are just jumping on the bandwagon with a view to publishing material without sitting back and carefully reviewing the value of these new tools. It seems sensible to me that we should learn what can and cannot be done with robots and then begin to integrate them into analytical laboratories to maximize their potential. Simply mechanizing manual processes using robots offers a solution to hard-pressed managers forced to cut staffing levels and budgets, but the major benefits can only be accrued when a detailed specification of the analytical problem is outlined and then the best, most economic solution found to meet that objective. I do not want to discourage the use of laboratory robots, rather the reverse. Analytical chemists have much to gain from industrial design engineers with skills in the reproduction of robotics. However, these engineers know little if anything of the constraints put upon analytical chemists. The chemist, on the other hand, may well have been directed to use a particular analytical methodology for a number of reasons and he may have lost sight of the major objectives simply presenting how the job is actually carried out manually. A period of testing and of design specification to the precise requirements can often be useful prior to implementing a robotic approach.

In the April 1982 edition of *JAC* I wrote:

> An interesting development at Pittsburgh was the introduction of an automated system based on robotics. The Zymark Corporation was formed in March 81 to develop, manufacture and market instrumentation for automated laboratory-scale processes used in chemistry and biochemistry. The Zymate Laboratory Automation system combines robotics and laboratory stations to automate the procedures used in sample preparation. For example liquid handling, sample conditioning, separation and chemical modification. In addition to the instrumentation, the Zymark Corporation has developed an educational program to provide a foundation in modern techniques of sample preparation—this program is available on a subscription basis. Whilst the approach is not totally new—the Robot Chemist and similar systems were launched more than 10 years ago—the technology now available makes the chances of success of this new venture more likely. Future developments along similar lines are eagerly awaited.

In 1982 Zymate had made some obvious progress, and the interest shown in their projects was very high. In the UK several meetings have been held, mainly under the auspices of the Royal Society of Chemistry's Analytical Division and this clearly shows that several large companies and the various governmental organizations have and will continue to invest effort into robotics research. At the 5th Summer School on Automatic Chemistry we were fortunate enough to have a demonstration experiment controlling a simple robotic work-station from a microcomputer. Several tutorial sessions were provided on the use of robots and the attendance at these clearly showed the level of interest. No doubt in ensuing years the involvement in robotics will have to be increased significantly so that this course can keep pace with user groups' interests.

However, clearly not every group can afford the level of investment to carry out the necessary background research in robotics. An interesting development has been launched: the Low-Cost Robot Devices Club, which attempts to bring several users together to share investment and benefit from the experiences gained.

The Club, which is operated by Femmright Ltd (473 Staines Road, Bedfont, Feltham, Middlesex TW14 8BL, UK; tel.: 01 890 1645), brings together a group of academic and industrial organizations. The work programme of the club is based on various aspects of laboratory unit operations (for example weighing, grinding, manipulation, liquid handling, separation etc.). The aim is to set up automated feeding of analytical instruments by such methods as robotic loading of carousels or direct injection of liquids etc., the same automatic or robotic handling will move on or replace tubes, locators and other work-pieces. The integration of these operations and movements using computer software will also be designed to capture data, process it and display the results. The objective will be to make work-stations completely automated with alarms to be sounded if the process malfunctions. These automated techniques will relieve the laboratory staff from multiple tests and routine tasks. Such techniques will improve the quality and dependability of analytical results. In addition, these projects will include as a vital facet of their programme a financial costing of automated work-stations, which will be compared with a manual method. Towards these ends the projects will be systematically and scientifically aimed. This approach should ensure that robotics is introduced for fully justifiable reasons into laboratory life.

Two projects are already in operation. The first is concerned with weighing, the second with measurement. The first has the Laboratory of the Government Chemist as a collaborator and is designed to measure biomass in fermentation broths in batches of 50 to 60 samples/day using automated or robotic methods.

In the past, Club projects of this type have been successful, albeit over a short period whilst the participants have an initial enthusiasm. How this one progresses will be watched with interest. Full details of the costs of Club involvement are available from Femmright; the Club is open both to UK companies and those outside.

Whilst there is a degree of similarity between the Club approach and that taken by Zymate it will be interesting to see how each set of analytical instrumentation develops. By opening its doors outside of the UK Femmright will have a larger market to aim for and thus a better chance of survival. Hopefully both groups will thrive and benefit from healthy competition.

Any developments along these lines in other countries would make interesting reading. So if you know of or are associated with developments in robotics why not write to me on the subject?

**Peter B. Stockwell**

